# Multiple Biomarkers and Atrial Fibrillation in the General Population

**DOI:** 10.1371/journal.pone.0112486

**Published:** 2014-11-17

**Authors:** Renate B. Schnabel, Philipp S. Wild, Sandra Wilde, Francisco M. Ojeda, Andreas Schulz, Tanja Zeller, Christoph R. Sinning, Jan Kunde, Karl J. Lackner, Thomas Munzel, Stefan Blankenberg

**Affiliations:** 1 Department of General and Interventional Cardiology, University Heart Center Hamburg-Eppendorf, Germany; 2 Department of Medicine 2, University Medical Center of the Johannes Gutenberg-University Mainz, Germany; 3 Center of Thrombosis and Hemostasis University Medical Center of the Johannes Gutenberg-University Mainz, Germany; 4 BRAHMS GmbH, Hennigsdorf/Germany; 5 Institute of Clinical Chemistry and Laboratory Medicine, University Medical Center of the Johannes Gutenberg-University Mainz, Germany; Inserm, France

## Abstract

**Background:**

Different biological pathways have been related to atrial fibrillation (AF). Novel biomarkers capturing inflammation, oxidative stress, and neurohumoral activation have not been investigated comprehensively in AF.

**Methods and Results:**

In the population-based Gutenberg Health Study (n = 5000), mean age 56±11 years, 51% males, we measured ten biomarkers representing inflammation (C-reactive protein, fibrinogen), cardiac and vascular function (midregional pro adrenomedullin [MR-proADM], midregional pro atrial natriuretic peptide [MR-proANP], N-terminal pro-B-type natriuretic peptide [Nt-proBNP], sensitive troponin I ultra [TnI ultra], copeptin, and C-terminal pro endothelin-1), and oxidative stress (glutathioneperoxidase-1, myeloperoxidase) in relation to manifest AF (n = 161 cases). Individuals with AF were older, mean age 64.9±8.3, and more often males, 71.4%. In Bonferroni-adjusted multivariable regression analyses strongest associations per standard deviation increase in biomarker concentrations were observed for the natriuretic peptides Nt-proBNP (odds ratio [OR] 2.89, 99.5% confidence interval [CI] 2.14–3.90; *P*<0.0001), MR-proANP (OR 2.45, 99.5% CI 1.91–3.14; *P*<0.0001), the vascular function marker MR-proADM (OR 1.54, 99.5% CI 1.20–1.99; *P*<0.0001), TnI ultra (OR 1.50, 99.5% CI 1.19–1.90; P<0.0001) and. fibrinogen (OR 1.44, 99.5% CI 1.19–1.75; P<0.0001). Based on a model comprising known clinical risk factors for AF, all biomarkers combined resulted in a net reclassification improvement of 0.665 (99.3% CI 0.441–0.888) and an integrated discrimination improvement of >13%.

**Conclusions:**

In conclusion, in our large, population-based study, we identified novel biomarkers reflecting vascular function, MR-proADM, inflammation, and myocardial damage, TnI ultra, as related to AF; the strong association of natriuretic peptides was confirmed. Prospective studies need to examine whether risk prediction of AF can be enhanced beyond clinical risk factors using these biomarkers.

## Introduction

With an increasing prevalence atrial fibrillation (AF) and its sequelae have become a significant public health burden not only through rising costs [1], [2]. Despite its clinical relevance the pathophysiological background of atrial remodeling and AF is little understood. Several biological pathways have been studied in depth to gain insights into disease susceptibility. Most consistently three pathways have been focused on: inflammation, oxidative stress and neurohumoral activity [3]–[5]. Inflammatory changes are present in atrial tissue specimens even in lone AF patients without overt cardiovascular disease. [6] Besides inflammation, signs of oxidative stress are omnipresent in AF. Myocardial nicotinamide adenine dinucleotide phosphate oxidase activity and, to a minor extent, uncoupling of the endothelial nitric oxide synthase generate reactive oxygen species in atria of patients with AF. [7] In experimental studies anti-oxidant ascorbate prevents AF by reducing oxidative stress and consecutive atrial remodeling [5]. Furthermore, autonomous imbalance and enhanced neurohumoral activation are risk factors for AF and perpetuate disease. Atrial and B-type natriuretic peptides and their precursors are cardiac specific markers of cardiovascular stress [4], [8].

Recent investigations showed associations of circulating inflammatory biomarkers, oxidative stress and natriuretic peptides with AF risk that have not entered clinical practice yet [9]–[12]. In the meantime, novel blood biomarkers that reflect the three major pathophysiological pathways of AF have been reported. We hypothesized that newer biomarkers of cardiac and vascular function in normal physiology and during vascular stress (mid-regional pro adrenomedullin [MR-proADM], Copeptin, C-terminal pro endothelin-1) [13], sensitve cardiac troponin I ultra [TnI ultra] [14], and oxidative stress (glutathioneperoxidase-1, myeloperoxidase) [15] may be more strongly correlated with AF in a contemporary population-based cohort in comparison with known markers such as C-reactive protein [CRP], fibrinogen, and the natriuretic peptide precursor N-terminal pro B-type natriuretic peptide [Nt-proBNP] [9]–[13].

## Methods

### Ethics statement

Prior to enrolment participants signed written, informed consent. The study has been approved by the local Ethics Committee (Landesaerztekammer Rheinland-Pfalz, 837.020.07).

### Study participants

Current analyses are based on the first 5000 individuals of the Gutenberg Health Study. The cohort constitutes a randomly selected population-based sample of European descent incepted in 2007 at the Department of Medicine 2, University Medical Center Mainz. Study participants are enrolled within 10-year age strata from 35–74 years. During a 5-hour clinic visit comprehensive information on cardiovascular risk factors are collected by standardized computer-assisted interview and anthropometric measures. Cardiovascular risk factor definitions comprised the following: smoking status comprised the categories non-smokers (never smokers and former smokers) and smokers. Diabetes mellitus was diagnosed when individuals reported a physician diagnosis of diabetes and/or a fasting blood glucose concentration of ≥126 mg/dL (minimum 8-hour fast) or a blood glucose level of ≥200 mg/dL at any time was measured on site. Dyslipidemia was defined based on a physician's diagnosis of dyslipidemia and/or an LDL/HDL ratio of >3.5. The definition of hypertension comprised anti-hypertensive drug treatment and/or a mean systolic blood pressure of ≥140 mmHg and/or a mean diastolic blood pressure of ≥90 mmHg. A history of cardiovascular disease (CVD) was self-reported myocardial infarction, stroke, prevalent coronary heart disease and heart failure. Heart failure was defined by clinic (New York Heart Association classification, heart failure medication) and echocardiography (left ventricular ejection fraction<55%).

The diagnosis of AF was made on a history of AF reported by the participant during the computer assisted interview and/or the ECG documentation of AF or atrial flutter [16]. AF was adjudicated by at least two physicians with cardiology training and experience in ECG reading. In two individuals the information on AF was missing.

### Biomarker determination

Routine laboratory parameters were measured from fasting blood samples by standardized methods for CRP, blood glucose, creatinine, fibrinogen and lipids at enrolment. For additional measurements, samples were aliquotted and stored at −80°C immediately after blood draw. In EDTA plasma, we measured Copeptin (functional assay sensitivity <1 pmoL/L), CT proendothelin-1 (functional assay sensitivity 19 pmoL/L), MR-proADM (functional assay sensitivity 0.25 nmoL/L), MR-proANP (functional assay sensitivity <10 pmoL/L) (Kryptor Immunoassays, B.R.A.H.M.S, GmbH, Germany), myeloperoxidase (intra–/inter-assay coefficient of variation 6.2/8.6) (CardioMPO kit, Prognostix, USA), and serum Nt-proBNP (intra–/inter-assay coefficient of variation 2.6/1.5) (Elecsys proBNP II Roche Diagnostics, Germany) biomarkers using commercially available assays. Sensitive TnI ultra was measured using Dimension RxL TnI (Siemens Healthcare Diagnostics, Germany) with a detection limit of 6 pg/mL and an assay range of 0–50.000 pg/mL. Glutathione-peroxidase-1 activity was determined in washed red cells obtained from whole blood anticoagulated with EDTA. Glutathione-peroxidase-1 was measured as previously described using the Ransel test kit (Randox, UK). [17].

### Statistical methods

Available case analysis was used. To give an impression of the representativeness of the sample of the underlying population we also provide the baseline characteristics weighted for the age and sex distribution of the general population (N = 210.867, ©Statistisches Bundesamt, Wiesbaden 2011). Skewed variables (|skewness|>1) including selected biomarkers were logarithmically transformed to achieve near normal distribution. Raw characteristics of the sample are given as mean and standard deviation for continuous variables, or median (25^th^ and 75^th^ percentile) for variables with a skewed distribution. Number and percent are shown for categorical variables. Characteristics are also shown weighted according to the age and sex distribution of the study population (N = 210,867, data of the German Federal Statistical Office, Wiesbaden, 2007). Sample quantiles were computed nonparametrically, except for those quantiles were the number of observations below the limit of detection did not permit this (CRP and TnI), in that case, a parametric estimate was used via Tobit regression after log-transformation using a t-distribution.

The panel of biomarkers was related to AF. In logistic regression models, biomarkers were tested for their association with AF per one standard deviation increase. Models were adjusted for age and sex as well as for age, sex and atrial fibrillation risk factors body mass index, systolic blood pressure, antihypertensive medication, and a history of cardiovascular disease. For these models the values CRP or TnI below the detection limit were substituted by the constant proposed by Richardson and Ciampi [18].

To understand the ranking of the biomarkers in their strength of association with AF, a classification tree was built for circulating markers that remained statistically significantly related to AF in multivariable-adjusted analyses. A tree is grown as follows: first the variable that best separates the data into two groups is used to split the data. Then this is repeated on each subgroup recursively until a stopping criterion is reached. The end result will be a model that is too complex and most likely overfits the data. To avoid this the tree is pruned using cross-validation [19].

We further assessed the area under the receiver operating characteristic curve and performed reclassification analyses [20] on models based on clinical variables of the Framingham risk score (age, age^2^, sex, male sex*age^2^, body mass index, systolic blood pressure, antihypertensive medication, congestive heart failure, congestive heart failure*age) [21] and biomarkers associated with AF in multivariable-adjusted analyses to assess the additive discriminative ability of the biomarkers between AF individuals and the rest of the sample for the biomarkers separately and in combination. Summary statistics of net reclassification improvement and integrated discrimination improvement were calculated.

We assumed a threshold of *P*<0.05 as statistically significant. To account for multiple testing we performed a Bonferroni correction for the number of tests applied in each analysis.

### Secondary analyses

In secondary analyses we performed logistic regression analyses for AF including left ventricular ejection fraction and serum creatinine as covariates. Further models were computed for those individuals with and without AF on the ECG at the time of blood draw separately.

For statistical calculations we used R software, Version 3.0.2 (R Development Core Team, 2009). R: A language and environment for statistical computing. R Core Team, Vienna, Austria. URL http://www.R-project.org).

## Results

The characteristics of the total sample and for individuals with AF are shown in **Table 1**. Whereas the mean age of individuals without AF was 55.2±10.9 years (49.9% female), participants with AF were about ten years older (64.9±8.3 years) and less likely to be female (28.6%). Data weighted for the age and sex distribution of the underlying population revealed similar results (**Table S1 in File S1**).

**Table 1:** Characteristics of the sample according to AF status.

Variable	Individuals without AF N = 4837	Individuals with AF N = 161
Age, years	55.2±10.9	64.9±8.3
Female sex, N (%)	2413 (49.9)	46 (28.6)
Current smoking, N (%)	941 (19.5)	18 (11.2)
Body mass index [kg/m^2^]	27.1±4.8	29.3±5.4
Height [m]	1.7±0.09	1.73±0.09
Systolic blood pressure [mmHg]	132.7±17.7	133.8±17.8
Diastolic blood pressure [mmHg]	83.2±9.4	82.4±11.1
Heart rate [bpm]	68.8±10.8	69.5±13.0
Total cholesterol [mg/dL]	223.9±41.2	211.0±44.0
HDL-cholesterol [mg/dL]	56.7±15.8	49.7±15.8
Diabetes, N (%)	353 (7.3)	21 (13.0)
Hypertension, N (%)	2445 (50.6)	118 (73.3)
Hypertension treatment, N (%)	1336 (27.6)	96 (59.6)
History of coronary artery disease, N (%)	192 (4)	34 (22.2)
History of myocardial infarction, N (%)	134 (2.8)	22 (13.8)
Prevalent heart failure, N (%)	887 (18.4)	78 (48.8)
*Biomarkers*		
Creatinine [mg/dL]	0.88 (0.79/0.97)	0.95 (0.83/1.06)
Glutathione-peroxidase-1 [U/gHb]	167.3±38.0	164.7±37.5
Myeloperoxidase [ng/mL]	299.3 (237.87/373.98)	323.24 (241.49/376.77)
C-reactive protein [mg/L]	1.6 (0.93*/3.2)	2.50 (1.37/4.93)
Fibrinogen [mg/dL]	345 (303/398)	404 (347/499)
MR-proADM [nmol/L]	0.46 (0.39/0.54)	0.60 (0.50/0.72)
MR-proANP [pmol/L]	65.2 (48.7/88.2)	134.6 (79.9/219.3)
Nt-proBNP [pg/mL]	60.21 (22. 72/118.21)	290.60 (90.44/977.73)
Copeptin [pmol/L]	2.75 (1.77/4.38)	3.87 (2.53/6.63)
CT-pro endothelin-1 [pmol/L]	58.7 (50.3/67.7)	69.9 (60.0/84.7)
TnI ultra [pg/mL]	5.3* (3.5*/8.0)	9.0 (6.0/15.0)

Provided are mean and standard deviation for continuous variables, or median (25^th^ and 75^th^ percentile) for variables with a skewed distribution (|skewness|>1). Number and percent are shown for categorical variables.

*Sample quantile estimated using parametric model.

Abbreviations: AF, atrial fibrillation; CT-pro-endothelin-1, C-terminal pro endothelin-1; HDL, high density lipoprotein; MR-proADM, mid-regional pro adrenomedullin; MR-proANP, midregional pro atrial natriuretic peptide; Nt-proBNP, N-terminal pro B-type natriuretic peptide; TnI ultra, sensitive troponin I ultra.

In individuals with AF classical risk factor burden was higher. Prevalent cardiovascular disease was also more frequent in AF with a history of coronary artery disease in about one fifth of the subgroup, myocardial infarction in approximately 14% compared to a prevalence of less than 5% in participants without AF. Nearly half of the patients with AF had prevalent heart failure. Creatinine concentrations were higher in AF individuals 0.95 mg/dL (0.83/1.06 mg/dL) compared to the non-AF individuals 0.88 mg/dL (0.79/0.97 mg/dL). Most biomarker distributions appeared to be higher in AF. There seemed to be greater systemic inflammatory activity reflected by increased CRP and fibrinogen concentrations, whereas the distributions of glutathione-peroxidase-1 and myeloperoxidase largely overlapped with values in the overall cohort. All biomarkers of cardiovascular function were elevated.

Spearman partial correlation coefficients with AF revealed highest estimates for MR-proANP (r = 0.21), Nt-proBNP (r = 0.20), fibrinogen (r = 0.13), and MR-proADM (r = 0.12) after accounting for age and sex (**Table S2 in File S1**).

**Figure 1:**
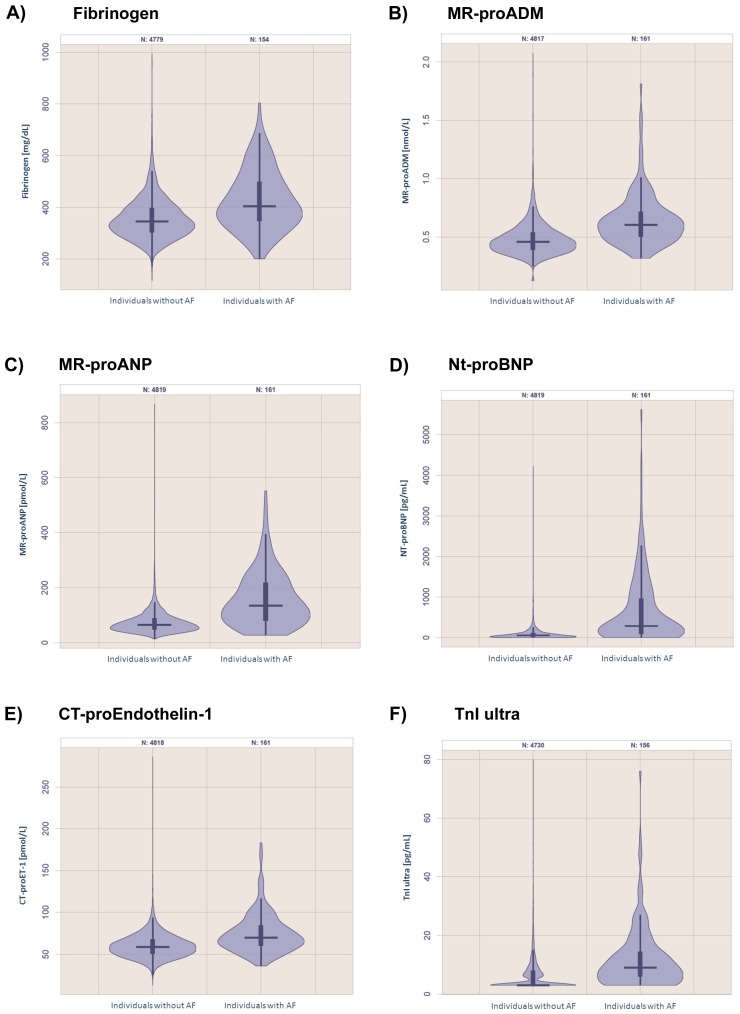
Violin plots of the distribution of circulating biomarkers in the total sample and in individuals with AF for markers that remained statistically significant in relation to AF in multivariable-adjusted models. For presentational reasons some outliers were removed from the plots. For MR-proADM values above 2.5 nmol/L (N = 2), Nt-proBNP values above 8000 pg/mL (N = 3), TnI ultra values above 80 pg/mL (N = 12) were excluded. Abbreviations: AF, atrial fibrillation; CT-proET, CT-pro endothelin-1; MR-proADM, mid-regional pro adrenomedullin; MR-proANP, midregional pro atrial natriuretic peptide; N-terminal pro B-type natriuretic peptide; TnI, troponin.

The panel of biomarkers was significantly associated with AF, P<0.0001. In Bonferroni-adjusted multivariable linear regression analyses greatest odds ratios [OR] per standard deviation increase in biomarker concentrations were observed for the natriuretic peptides Nt-proBNP (OR 2.89, 99.5% confidence interval [CI] 2.14–3.90; *P*<0.0001), MR-proANP (OR 2.45, 99.5% CI 1.91–3.14; *P*<0.0001) and the vascular function marker MR-proADM (OR 1.54, 99.5% CI 1.20–1.99; *P*<0.0001) (**Table 2**). The inflammatory biomarker fibrinogen remained related to AF (OR 1.44, 95% CI 1.19–1.75; *P*<0.0001) whereas CRP lost statistical significance, *P* = 1.00 in the multivariable model. Violin plots of the distribution of circulating biomarkers in the total sample and in individuals with AF for markers that retained statistical significance in multivariable-adjusted models are provided in **Figure 1**.

**Table 2:** Multivariable logistic regression models for biomarkers in relation to AF.

Variable	Odds Ratio per Standard Deviation	99.5% Confidence Interval		*P* Value*
Glutathione-peroxidase-1 [U/gHb]	0.88	0.70	1.12	1.00
	0.85	0.67	1.08	0.61
Myeloperoxidase [ng/mL]	1.09	0.87	1.36	1.00
	1.01	0.80	1.27	1.00
C-reactive protein [mg/L]	1.26	1.02	1.55	0.023
	1.11	0.88	1.39	1.00
Fibrinogen [mg/dL]	1.60	1.33	1.92	<0.0001
	1.44	1.19	1.75	<0.0001
MR-proADM [nmol/L]	1.86	1.48	2.34	<0.0001
	1.54	1.20	1.99	<0.0001
MR-proANP [pmol/L]	2.69	2.12	3.42	<0.0001
	2.45	1.91	3.14	<0.0001
Nt-proBNP [pg/mL]	3.36	2.52	4.49	<0.0001
	2.89	2.14	3.90	<0.0001
Copeptin [pmol/L]	1.27	1.00	1.61	0.049
	1.17	0.92	1.50	0.70
CT-pro endothelin-1 [pmol/L]	1.70	1.36	2.12	<0.0001
	1.43	1.14	1.80	0.00011
TnI ultra [pg/mL]	1.61	1.29	2.00	<0.0001
	1.50	1.19	1.90	<0.0001

*P values were Bonferroni corrected for ten tests. Multivariable-adjustment included age, sex (upper row) and age, sex, body mass index, systolic blood pressure, antihypertensive medication, and a history of cardiovascular disease (lower row). Biomarkers were logarithmically transformed except for glutathione-peroxidase-1 and fibrinogen.

Abbreviations: AF, atrial fibrillation; CT-pro-endothelin-1, C-terminal pro endothelin-1; TnI ultra, sensitive troponin I ultra; MR-proADM, mid-regional pro adrenomedullin; MR-proANP, midregional pro atrial natriuretic peptide; Nt-proBNP, N-terminal pro B-type natriuretic peptide.

Associations were stronger in individuals in whom AF was present at the time of blood draw (**Table S3 in File S1**). Associations did not change markedly when adjusted for left ventricular ejection fraction and creatinine concentrations (**Table S4 in File S1**). Analyses stratified by heart failure status revealed similar results in individuals without heart failure compared to participants with manifest disease (**Table S5 in File S1**).

**Figure 2:**
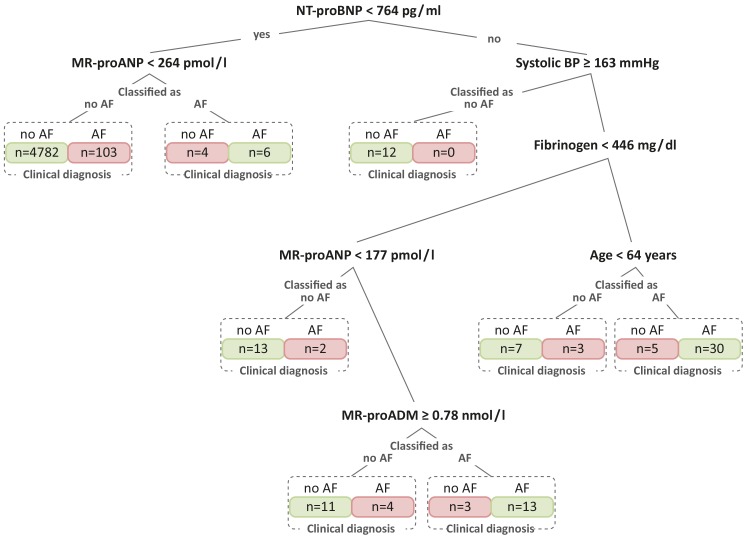
Regression tree for biomarkers that remained statistically significant in relation to AF in multivariable models. Provided are the mean that was selected for the split of the tree and the number of individuals for the respective branches of the regression tree. For every branch the classification of individuals according to the model and the correct, clinical diagnosis are shown. In red the number of participants misclassified by the statistical model is indicated. In two individuals the information on AF was missing. Abbreviations: AF, atrial fibrillation; BP, blood pressure; MR-proADM, mid-regional pro adrenomedullin; MR-proANP, midregional pro atrial natriuretic peptide; N-terminal pro B-type natriuretic peptide; TnI, troponin.

In classification and regression tree analyses comparing the selection of clinical risk factors and biomarkers, which were significantly related to AF in multivariable-adjusted models, the natriuretic peptides Nt-proBNP and MR-proANP were the biomarkers selected by the model for the first two branchings of the tree, together with systolic blood pressure (**Figure 2**). The variables with the best discriminatory ability were selected first by the models and represent the basis of the tree. After systolic blood pressure, fibrinogen, age entered the tree besides MR-proANP a second time. MR-proADM was also selected to further split the tree. The natriuretic peptides thus seemed to provide the greatest gain in information to discern AF from non-AF participants. The strongest clinical indicators for AF were systolic blood pressure and age.

Reclassification analyses based on clinical risk factors and the strongest biomarkers in relation to AF in multivariable analyses revealed significant reclassification for all biomarkers when added to the variables used in the Framingham risk model (**Table 3**). Greatest NRI and IDI was observed for the natriuretic peptides (NRI MR-proANP 0.599, IDI 0.088; NRI Nt-proBNP 0.545, IDI 0.095). Largest increases in the area under the curve (AUC) for the basic model 0.82 (99.3% CI 0.77–0.87) were observed for the addition of natriuretic peptides MR-proANP (AUC 0.85, 99.3% CI 0.80–0.90) or Nt-proBNP to the model (AUC 0.84, 99.3% CI 0.79–0.90). MR-proADM and TnI ultra were less strong and increased the AUC only to 0.83. All biomarkers combined resulted in a NRI of 0.665 (99.3% CI 0.441–0.888) with an IDI of more than 13% and an AUC of 0.86 (99.3% CI 0.80–0.91). The combination of all biomarkers provided more information than any of the biomarkers separately. R^2^ reached 0.316 compared to 0.193 in a model containing the risk factors only.

**Table 3:** Reclassification analysis for biomarkers significantly related to AF in multivariable-adjusted models.

	Odds Ratio per SD	*P* Value	NRI	*P* Value	IDI	*P* Value	Area Under the Curve	R^2^
Model with Framingham risk score variables	–	–	–	–	–	–	0.82 (0.77,0.87)	0.193
Fibrinogen	1.46 (1.21, 1.76)	<0.0001	0.345 (0.123, 0.566)	0.00020	0.023 (0.008, 0.038)	0.00031	0.82 (0.77,0.87)	0.213
MR-proADM	1.55 (1.21, 1.98)	<0.0001	0.212 (−0.005, 0.429)	0.060	0.014 (0.002, 0.027)	0.013	0.83 (0.78,0.87)	0.211
MR-proANP	2.53 (1.99, 3.22)	<0.0001	0.599 (0.382, 0.815)	<0.0001	0.088 (0.056, 0.120)	<0.0001	0.85 (0.80,0.90)	0.284
Nt-proBNP	3.04 (2.27, 4.07)	<0.0001	0.545 (0.328, 0.762)	<0.0001	0.095 (0.063, 0.127)	<0.0001	0.84 (0.79,0.90)	0.286
CT-pro endothelin-1	1.44 (1.15, 1.80)	<0.0001	0.158 (−0.059, 0.375)	0.35	0.014 (0.003, 0.026)	0.0070	0.82 (0.78,0.87)	0.209
TnI ultra	1.27 (1.11, 1.46)	<0.0001	0.500 (0.281, 0.720)	<0.0001	0.015 (0.004, 0.025)	0.00023	0.83 (0.79,0.88)	0.217
**All biomarkers combined**			**0.665 (0.441, 0.888)**	**<0.0001**	**0.125 (0.084, 0.166)**	**<0.0001**	**0.86 (0.81,0.91)**	**0.315**

The baseline model comprised Framingham risk score variables [21]: age, age^2^, sex, male sex*age^2^, body mass index, systolic blood pressure, antihypertensive medication, congestive heart failure, congestive heart failure*age. All biomarkers except for fibrinogen were logarithmically transformed for analyses. *P* values were Bonferroni corrected for seven tests.

R^2^ is Nagelkerke R^2^. Odds ratio, NRI, IDI and AUC are presented with 99.3% confidence interval in brackets.

**Abbreviations:** AF, atrial fibrillation; AUC, area under the curve; CRP, C-reactive Protein; CT-pro-endothelin-1, C-terminal pro endothelin-1; TnI ultra, sensitive troponin I ultra; IDI, integrated discrimination improvement; MR-proADM, mid-regional pro adrenomedullin; MR-proANP, midregional pro atrial natriuretic peptide; NRI, net reclassification improvement; Nt-proBNP, N-terminal pro B-type natriuretic peptide.

## Discussion

In our population-based sample, we identified biomarkers of cardiovascular function, MR-proADM and CT-pro endothelin, as novel correlates of AF. Natriuretic peptides were confirmed as strong predictors of AF. TnI ultra as an indicator of myocardial damage was also significantly related to AF. CRP lost statistical significance in relation to AF in multivariable-adjusted models whereas the pro-inflammatory and hemostatic biomarker fibrinogen remained significantly associated. No relevant association was observed for the examined indicators of oxidative stress. The increase in the AUC and net reclassification for biomarkers separately or combined was significant, but remained moderate.

As expected, natriuretic peptides were the strongest correlates of AF. However, we know that the additional value of B-type natriuretic peptide for reclassification in addition to clinical and electrocardiographic risk indicators may be modest [12]. More novel biomarkers such as MR-proADM and CT-pro-endothelin have been in the focus of interest in cardiovascular disease because they reflect the activity of hormones central to vascular homeostasis [22], [23]. In contrast to adrenomedullin and endothelin, the hormone precursors show higher analyte stability than their short-lived active hormones and more reliable assay characteristics [24], [25].

Adrenomedullin exerts beneficial long-lasting vasodilatory and blood pressure lowering effects. It is expressed in endothelial cells but can also be found in the myocardium where adrenomedullin specific binding sites are expressed [26], [27]. Whereas adrenomedullin has consistently been reported as a biomarker in a variety of diseases among them coronary artery disease and heart failure [28], [29], little is known about the peptide in AF. In normal human cardiac tissue, the atria are the main site for positive inotropic effects of adrenomedullin measured by an increased force of contraction. Effects are blunted in ventricular myocardium and the failing heart [30]. During atrial stretch the adrenomedullin signaling cascade is down-regulated which may enhance susceptibility to AF [31]. In human cohorts MR-proADM appears to be less well suited to predict recurrence of AF [13] or incident AF events. [32] Whether MR-proADM is related to outcome in AF as shown for congestive heart failure patients remains to be elucidated [29].

In contrast, endothelin-1 is one of the most potent vasoconstrictors, but also exerts hormone-like activities. Compensatory elevation of endothelin-1 activity can be measured in heart failure and AF [33]. More important than systemic endothelin may be the local endothelin production and auto- and paracrine effects in the atria in AF. In atrial tissue of patients with AF distinct gene expression patterns of the endothelin system can be demonstrated [34]. Changes seem to be pronounced in persistent AF compared to paroxysmal types of AF. Acute and chronic stretch response of the atria results in increased endothelin expression [31]. Atrial endothelin actions comprise the induction of natriuretic peptide secretion and increased mechanical and receptor-mediated transcriptional activation in cardiac myocytes [35]. The endothelin receptor antagonist bosentan attenuates transcription factor activity [31]. Whereas a small study that measured endothelin-1 did not show an association of the hormone with AF [36], systemic CT-pro-endothelin-1 elevation has been observed in cardiovascular disease and in patients with manifest AF compared to patients in sinus rhythm with a history of AF [13], [23]. The origin of the measured circulating CT-pro-endothelin-1 is less clear. Endothelin-1 is most abundantly produced by vascular endothelium, but may also be a spill-over of cardiac myocyte or fibroblast endothelin-1 generation under the conditions of AF. [37].

For both vascular biomarkers we can extend recent findings of elevated MR-proADM and CT-pro-endothelin concentrations in patients with AF towards the general population [13].

Copeptin as a precursor of vasopressin only showed a borderline association with AF and lost statistical significance after risk factor adjustment similarly to recent publications [13], [32].

Cardiac troponins are specific markers of myocardial injury. Tachyarrhythmias can be accompanied by elevated troponin despite the absence of coronary disease [38]. Minor troponin elevations are observed frequently in patients admitted with AF and may have prognostic importance [14]. Troponin T has been related to early recurrence of AF episodes in AF patients [13]. In our study in ambulatory individuals from the general population, TnI ultra was also significantly increased in AF compared to participants free of manifest disease although it was not selected among the strongest biomarkers in regression tree analyses. With the advent of more sensitive assays troponins will become increasingly common in biomarker panels assessing cardiovascular disease. More data will be accrued to understand the role of troponin elevations and outcome in AF. We will then get a better understanding of whether these biomarkers separately or in combination permit better risk prediction and may highlight pathways that can be addressed for therapeutic intervention.

Most of the strongest biomarkers in association with AF such as the natriuretic peptides, troponins and CT-pro-endothelin have also been related to heart failure [39]–[42]. Heart failure is an underlying disease in up to 50 percent of AF patients [43] and observed biomarker associations may be due to heart failure. However, despite a prevalence of almost 50 percent of heart failure in participants with AF in our cohort, association results did not markedly differ when analyses were stratified by heart failure status. To further elucidate the impact of heart failure on biomarker concentrations in AF larger cohorts or clinical AF cohorts might be necessary.

Despite sound experimental evidence on the central role of oxidative stress in AF genesis and perpetuation [44], we failed to show meaningful associations of circulating indicators of oxidative stress burden, i.e. glutathione-peroxidase-1 and myeloperoxidase. The missing correlation of systemically measured biomarkers is consistent with recent findings for different markers of oxidative stress myeloperoxidase and homocysteine [11], [12]. One reason for the discrepancy may be the blood measurement that reflects systemic levels of biomarkers and may not adequately reflect the local milieu in the atria. Similarly, inflammatory activity is highly enhanced in atrial tissue, but systemic C-reactive protein is only mildly elevated and does not relevantly improve risk prediction of AF whereas the inflammatory and hemostatic biomarker fibrinogen ranges among the strongest correlates of AF [11], [12].

### Limitations

Owing to the nature of the study design we cannot exclude reverse causation with biomarker changes secondary to disease onset. Prospective studies are needed to understand the benefit of biomarker determination for AF risk prediction in addition to known risk factors of incident AF in the general population [12]. Among the strengths of the study are the comparatively large sample size and the availability of a broad spectrum of reliably measurable known and new biomarkers in a contemporary cohort. The direct clinical impact of our study is limited. Our results are hypothesis generating and may encourage future investigations into the pathophysiology and predictive value of biomarkers reflecting vascular function for risk stratification and opportunities for intervention. Whether the determination of biomarkers separately or in a panel combining the strongest biomarkers can improve clinical care and outcome needs to be shown.

In conclusion, besides the natriuretic peptides MR-proANP and Nt-proBNP and fibrinogen we identified novel candidate biomarkers reflecting vascular function, MR-proADM and C-terminal pro endothelin-1, and myocardial damage, TnI ultra, in relation to AF in the general population that may improve risk assessment in AF and merit prospective investigation in future studies.

## Supporting Information

File S1 **Supporting tables. Table S1.** Characteristics of the sample according to AF status, weighted according to the age and sex distribution in the study population (N = 210,867). **Table S2.** Partial Spearman rank correlation coefficients for blood biomarkers and atrial fibrillation, adjusted for age and sex. **Table S3.** Logistic regression models for biomarkers in relation to history of AF in individuals with AF on the study ECG at the time of blood draw. **Table S4.** Logistic regression models for biomarkers in relation to AF adjusted for creatinine and left ventricular ejection fraction. **Table S5.** Age- and sex-adjusted logistic regression models for biomarkers in relation to AF stratified by heart failure status.
(DOCX)
