# Supplementary material for: Multiple Biomarkers and Atrial Fibrillation in the General Population
Source: PLoS One. 2014 Nov 17;9(11):e112486. doi: 10.1371/journal.pone.0112486 (PMC4234420; doi:10.1371/journal.pone.0112486)
Supplement: File S1 — Supporting tables. Table S1. Characteristics of the sample according to AF status, weighted according to the age and sex distribution in the study population (N = 210,867). Table S2. Partial Spearman rank correlation coefficients for blood biomarkers and atrial fibrillation, adjusted for age and sex. Table S3. Logistic regression models for biomarkers in relation to history of AF in individuals with AF on the study ECG at the time of blood draw. Table S4. Logistic regression models for biomarkers in relation to AF adjusted for creatinine and left ventricular ejection fraction. Table S5. Age- and sex-adjusted logistic regression models for biomarkers in relation to AF stratified by heart failure status. (DOCX) [file pone.0112486.s001.docx]

**Supporting Information**

**Table S1.** Characteristics of the sample according to AF status, weighted according to the age and sex distribution in the study population (N=210,867)

| **Variable** | **Individuals without Atrial Fibrillation**  **N=4837** | **Individuals with Atrial Fibrillation**  **N=161** |
| --- | --- | --- |
| Age, years | 51.9±11 | 63.2±9.5 |
| Female sex, % | 50.6 | 31.8 |
| Current smoking, N % | 21.2 | 11.6 |
| Body mass index [kg/m²] | 26.9±4.8 | 29.4±5.3 |
| Height [m] | 1.71±0.09 | 1.73±0.09 |
| Systolic blood pressure [mmHg] | 130.7±17 | 133±17.7 |
| Diastolic blood pressure [mmHg] | 83±9.3 | 82.5±11 |
| Heart rate [bpm] | 69±10.6 | 69.4±12.7 |
| Total cholesterol [mg/dL] | 221.8±40.7 | 211.9±43.9 |
| HDL-cholesterol [mg/dL] | 56.4±15.7 | 49.7±15.4 |
| Diabetes, % | 5.8 | 12.2 |
| Hypertension, % | 44.7 | 71.6 |
| Hypertension treatment, % | 22.9 | 57.7 |
| History of coronary artery disease, % | 2.9 | 21 |
| History of myocardial infarction, % | 2 | 12.7 |
| Prevalent heart failure, % | 17 | 46.3 |
| *Biomarkers* |  |  |
| Creatinine [mg/dL] | 0.87 (0.78, 0.97) | 0.93 (0.82, 1.05) |
| Glutathione-peroxidase-1 [U/gHb] | 165.8±37.6 | 165.2±38 |
| Myeloperoxidase [ng/mL] | 298.54 (238.71, 372.93) | 323.48 (238.23, 383.6) |
| C-reactive protein [mg/L] | 1.6 (0.5*, 3.1) | 2.3 (1.3, 4.71) |
| Fibrinogen [mg/dL] | 339 (298, 393) | 389 (336, 490) |
| MR-proADM [nmol/L] | 0.44 (0.38, 0.52) | 0.59 (0.48, 0.71) |
| MR-proANP [pmol/L] | 61.1 (45.5, 82) | 123.5 (72.1, 209.8) |
| Nt-proBNP [pg/mL] | 52.56 (23.06, 104.65) | 257.97 (64.99, 931.67) |
| Copeptin [pmol/L] | 2.62 (1.73, 4. 16) | 3.63 (2.45, 6.43) |
| CT-pro endothelin-1 [pmol/L] | 57 (49, 66.1) | 66.9 (58.1, 82.1) |
| TnI ultra [pg/mL] | 5* (3.4*, 7) | 9 (5.6*, 14) |

Provided are mean and standard deviation for continuous variables, or median (25^th^ and 75^th^ percentile) for variables with a skewed distribution (|skewness|>1). Percent is shown for categorical variables.

*Sample quantile estimated using parametric model.

Abbreviations: AF, atrial fibrillation; CT-pro-endothelin-1, C-terminal pro endothelin-1 ; HDL high density lipoprotein; MR-proADM, mid-regional pro adrenomedullin; MR-proANP, midregional pro atrial natriuretic peptide; Nt-proBNP, N-terminal pro B-type natriuretic peptide; TnI ultra, sensitive troponin I ultra.

**Table S2.** Partial Spearman rank correlation coefficients for blood biomarkers and atrial fibrillation, adjusted for age and sex

| **Variable** | **Total**  **Atrial fibrillation**  **N=161** | **Atrial fibrillation  on ECG**  **N=65** |
| --- | --- | --- |
| Glutathione-peroxidase 1 [U/gHb] | -0.023  *P*=0.099 | 0.00061  *P*=0.97 |
| Myeloperoxidase [ng/mL] | 0.017  *P*=0.24 | 0.012  *P*=0.41 |
| C-reactive protein [mg/L] | 0.046  *P*=0.0012 | 0.037  *P*=0.0096 |
| Fibrinogen [mg/dL] | 0.13  *P*<0.0001 | 0.14  *P*<0.0001 |
| Midregional-pro adrenomedullin [nmol/L] | 0.12  *P*<0.0001 | 0.13  *P*<0.0001 |
| Midregional-pro atrial natriuretic peptide [pmol/L] | 0.21  *P*<0.0001 | 0.25  *P*<0.0001 |
| N-terminal pro B-type natriuretic peptide [pg/mL] | 0.20  *P*<0.0001 | 0.24  *P*<0.0001 |
| Copeptin [pmol/L] | 0.043  *P*=0.0027 | 0.061  *P*<0.0001 |
| C-terminal-pro endothelin-1 [pmol/L] | 0.11  *P*<0.0001 | 0.12  *P*<0.0001 |
| Sensitive Troponin I ultra [pg/mL] | 0.10  *P*<0.0001 | 0.12  *P*<0.0001 |

Provided are Spearman rank correlation coefficients and respective *P* values. All biomarkers were logarithmically transformed for analyses aside from glutathione-peroxidase and fibrinogen.

**Table S3.**

Logistic regression models for biomarkers in relation to history of AF in individuals with AF on the study ECG at the time of blood draw

|  | **Individuals with AF on the Study ECG** | | | | **Individuals without AF on the Study ECG** | | | |
| --- | --- | --- | --- | --- | --- | --- | --- | --- |
| **Variable** | **Odds Ratio per Standard Deviation** | **99.5 %Confidence Interval** | | ***P* Value*** | **Odds Ratio per Standard Deviation** | **99.5%Confidence Interval** | | ***P* Value*** |
| Glutathione-peroxidase 1 [U/gHb] | 1.39 | 0.62 | 3.10 | 1.0 | 0.80 | 0.58 | 1.09 | 0.40 |
|  | 1.47 | 0.60 | 3.61 | 1.0 | 0.77 | 0.56 | 1.05 | 0.19 |
| Myeloperoxidase [ng/mL] | 1.19 | 0.52 | 2.70 | 1.0 | 1.09 | 0.81 | 1.45 | 1.00 |
|  | 1.24 | 0.50 | 3.08 | 1.0 | 1.03 | 0.77 | 1.38 | 1.00 |
| C-reactive protein [mg/L] | 1.51 | 0.64 | 3.54 | 1.0 | 1.20 | 0.92 | 1.58 | 0.52 |
|  | 1.85 | 0.68 | 5.05 | 0.85 | 1.10 | 0.83 | 1.48 | 1.00 |
| Fibrinogen [mg/dL] | 2.15 | 0.82 | 5.63 | 0.26 | 1.31 | 1.03 | 1.66 | 0.018 |
|  | 2.61 | 0.84 | 8.09 | 0.17 | 1.20 | 0.93 | 1.57 | 0.47 |
| Midregional pro adrenomedullin [nmol/L] | 0.89 | 0.40 | 2.00 | 1.0 | 1.43 | 1.07 | 1.92 | 0.0049 |
|  | 1.05 | 0.40 | 2.74 | 1.0 | 1.23 | 0.88 | 1.72 | 0.88 |
| Midregional pro atrial natriuretic peptide [pmol/L] | 0.91 | 0.41 | 2.04 | 1.0 | 1.60 | 1.20 | 2.15 | <0.0001 |
|  | 0.86 | 0.36 | 2.06 | 1.0 | 1.45 | 1.08 | 1.94 | 0.0044 |
| N-terminal pro B-type natriuretic peptide [pg/mL] | 1.51 | 0.67 | 3.39 | 1.0 | 1.61 | 1.14 | 2.28 | 0.0012 |
|  | 1.66 | 0.66 | 4.18 | 1.0 | 1.41 | 0.99 | 1.99 | 0.059 |
| Copeptin [pmol/L] | 0.79 | 0.35 | 1.78 | 1.0 | 1.04 | 0.76 | 1.42 | 1.00 |
|  | 1.03 | 0.40 | 2.69 | 1.0 | 1.00 | 0.73 | 1.37 | 1.00 |
| C-terminal pro endothelin-1 [pmol/L] | 0.77 | 0.34 | 1.78 | 1.0 | 1.28 | 0.96 | 1.72 | 0.17 |
|  | 0.81 | 0.32 | 2.04 | 1.0 | 1.11 | 0.83 | 1.50 | 1.00 |
| Sensitive Troponin I ultra [pg/mL] | 0.62 | 0.26 | 1.47 | 1.0 | 1.28 | 0.96 | 1.70 | 0.16 |
|  | 0.56 | 0.20 | 1.56 | 1.0 | 1.19 | 0.88 | 1.61 | 1.00 |

*P values were Bonferroni corrected for ten tests. Multivariable-adjustment included age, sex (upper row) and age, sex, body mass index, systolic blood pressure, antihypertensive medication, and a history of cardiovascular diseases (lower row). Biomarkers were logarithmically transformed for analyses except for glutathione-peroxidase-1 and fibrinogen.

**Table S4.**

Logistic regression models for biomarkers in relation to AF adjusted for creatinine and left ventricular ejection fraction

| **Variable** | **Odds Ratio per Standard Deviation** | **99.5% Confidence Interval** | | ***P* Value*** |
| --- | --- | --- | --- | --- |
| Glutathione-peroxidase 1 [U/gHb] | 0.88 | 0.69 | 1.12 | 1.0 |
| Myeloperoxidase [ng/mL] | 1.06 | 0.85 | 1.33 | 1.0 |
| C-reactive protein [mg/L] | 1.23 | 0.99 | 1.52 | 0.064 |
| Fibrinogen [mg/dL] | 1.57 | 1.30 | 1.89 | <0.0001 |
| Midregional pro adrenomedullin [nmol/L] | 2.05 | 1.54 | 2.72 | <0.0001 |
| Midregional pro atrial natriuretic peptide [pmol/L] | 2.73 | 2.09 | 3.56 | <0.0001 |
| N-terminal pro B-type natriuretic peptide [pg/mL] | 3.38 | 2.45 | 4.67 | <0.0001 |
| Copeptin [pmol/L] | 1.20 | 0.93 | 1.55 | 0.42 |
| C-terminal pro endothelin-1 [pmol/L] | 1.70 | 1.31 | 2.21 | <0.0001 |
| Sensitive Troponin I ultra [pg/mL] | 1.48 | 1.18 | 1.87 | <0.0001 |

*P* values were Bonferroni corrected for ten tests. Multivariable-adjustment included age, sex, creatinine and left ventricular ejection fraction. Biomarkers were logarithmically transformed for analyses except for glutathione-peroxidase and fibrinogen.

**Table S5.**

Age- and sex-adjusted logistic regression models for biomarkers in relation to AF stratified by heart failure status

| 1. **Individuals without heart failure**   **N=4019**  **Variable** | **Odds Ratio per Standard Deviation** | **99.5% Confidence Interval** | | ***P* Value*** |
| --- | --- | --- | --- | --- |
| Glutathione-peroxidase 1 [U/gHb] | 0.83 | 0.60 | 1.15 | 1.0 |
| Myeloperoxidase [ng/mL] | 1.02 | 0.74 | 1.40 | 1.0 |
| C-reactive protein [mg/L] | 0.96 | 0.87 | 1.57 | 1.0 |
| Fibrinogen [mg/dL] | 1.53 | 1.19 | 1.95 | <0.0001 |
| Midregional pro adrenomedullin [nmol/L] | 1.59 | 1.16 | 2.20 | 0.00044 |
| Midregional pro atrial natriuretic peptide [pmol/L] | 2.75 | 1.98 | 3.81 | <0.0001 |
| N-terminal pro B-type natriuretic peptide [pg/mL] | 3.24 | 2.14 | 4.93 | <0.0001 |
| Copeptin [pmol/L] | 1.26 | 0.91 | 1.76 | 0.46 |
| C-terminal pro endothelin-1 [pmol/L] | 1.47 | 1.06 | 2.05 | 0.010 |
| Sensitive troponin I ultra [pg/mL] | 1.31 | 1.02 | 1.85 | 0.025 |

| 1. **Individuals with heart failure**   **N=965**  **Variable** | **Odds Ratio per Standard Deviation** | **99.5% Confidence Interval** | | ***P* Value*** |
| --- | --- | --- | --- | --- |
| Glutathione-peroxidase 1 [U/gHb] | 0.97 | 0.67 | 1.40 | 1.0 |
| Myeloperoxidase [ng/mL] | 1.08 | 0.78 | 1.51 | 1.0 |
| C-reactive protein [mg/L] | 0.96 | 0.83 | 1.65 | 1.0 |
| Fibrinogen [mg/dL] | 1.57 | 1.15 | 2.14 | 0.00054 |
| Midregional pro adrenomedullin [nmol/L] | 1.77 | 1.22 | 2.57 | 0.00014 |
| Midregional pro atrial natriuretic peptide [pmol/L] | 2.50 | 1.69 | 3.71 | <0.0001 |
| N-terminal pro B-type natriuretic peptide [pg/mL] | 3.05 | 1.94 | 4.80 | <0.0001 |
| Copeptin [pmol/L] | 1.15 | 0.80 | 1.65 | 1.0 |
| C-terminal pro endothelin-1 [pmol/L] | 1.68 | 1.19 | 2.36 | 0.00022 |
| Sensitive troponin I ultra [pg/mL] | 1.31 | 1.18 | 2.66 | 0.00085 |

**P* values were Bonferroni corrected for ten tests. Biomarkers were logarithmically transformed for analyses except for glutathione-peroxidase and fibrinogen.
